# A Primer on the Analysis of High-Throughput Sequencing Data for Detection of Plant Viruses

**DOI:** 10.3390/microorganisms9040841

**Published:** 2021-04-14

**Authors:** Denis Kutnjak, Lucie Tamisier, Ian Adams, Neil Boonham, Thierry Candresse, Michela Chiumenti, Kris De Jonghe, Jan F. Kreuze, Marie Lefebvre, Gonçalo Silva, Martha Malapi-Wight, Paolo Margaria, Irena Mavrič Pleško, Sam McGreig, Laura Miozzi, Benoit Remenant, Jean-Sebastien Reynard, Johan Rollin, Mike Rott, Olivier Schumpp, Sébastien Massart, Annelies Haegeman

**Affiliations:** 1 Department of Biotechnology and Systems Biology, National Institute of Biology, Večna pot 111, 1000 Ljubljana, Slovenia; 2 Plant Pathology Laboratory, Université de Liège, Gembloux Agro-Bio Tech, TERRA, Passage des Déportés, 2, 5030 Gembloux, Belgium; lucie.tamisier@uliege.be (L.T.); johan.rollin@doct.uliege.be (J.R.); sebastien.massart@uliege.be (S.M.); 3 Fera Science Limited, York YO41 1LZ, UK; ian.adams@fera.co.uk (I.A.); Sam.McGreig@fera.co.uk (S.M.); 4 Institute for Agri-Food Research and Innovation, Newcastle University, King’s Rd, Newcastle Upon Tyne NE1 7RU, UK; neil.boonham@newcastle.ac.uk; 5 UMR 1332 Biologie du Fruit et Pathologie, INRA, University of Bordeaux, 33140 Villenave d’Ornon, France; thierry.candresse@inrae.fr (T.C.); marie.lefebvre@inra.fr (M.L.); 6 Institute for Sustainable Plant Protection, National Research Council, Via Amendola, 122/D, 70126 Bari, Italy; michela.chiumenti@ipsp.cnr.it; 7 Plant Sciences Unit, Flanders Research Institute for Agriculture, Fisheries and Food, Burg. Van Gansberghelaan 96, 9820 Merelbeke, Belgium; kris.dejonghe@ilvo.vlaanderen.be (K.D.J.); annelies.haegeman@ilvo.vlaanderen.be (A.H.); 8 International Potato Center (CIP), Avenida la Molina 1895, La Molina, Lima 15023, Peru; j.kreuze@cgiar.org; 9 Natural Resources Institute, University of Greenwich, Central Avenue, Chatham Maritime, Kent ME4 4TB, UK; G.Silva@greenwich.ac.uk; 10 Biotechnology Risk Analysis Programs, Biotechnology Regulatory Services, Animal and Plant Health Inspection Service, U.S. Department of Agriculture, Riverdale, MD 20737, USA; martha.m.wight@usda.gov; 11 Leibniz Institute-DSMZ, Inhoffenstrasse 7b, 38124 Braunschweig, Germany; Paolo.Margaria@dsmz.de; 12 Agricultural Institute of Slovenia, Hacquetova Ulica 17, 1000 Ljubljana, Slovenia; Irena.MavricPlesko@kis.si; 13 Institute for Sustainable Plant Protection, National Research Council of Italy (IPSP-CNR), Strada delle Cacce 73, 10135 Torino, Italy; laura.miozzi@ipsp.cnr.it; 14 ANSES Plant Health Laboratory, 7 Rue Jean Dixméras, CEDEX 01, 49044 Angers, France; benoit.remenant@anses.fr; 15 Agroscope, Route de Duillier 50, 1260 Nyon, Switzerland; jean-sebastien.reynard@agroscope.admin.ch (J.-S.R.); olivier.schumpp@agroscope.admin.ch (O.S.); 16 DNAVision, 6041 Charleroi, Belgium; 17 Sidney Laboratory, Canadian Food Inspection Agency, 8801 East Saanich Rd, North Saanich, BC V8L 1H3, Canada; mike.rott@canada.ca

**Keywords:** plant virus, high-throughput sequencing, bioinformatics, detection, discovery

## Abstract

High-throughput sequencing (HTS) technologies have become indispensable tools assisting plant virus diagnostics and research thanks to their ability to detect any plant virus in a sample without prior knowledge. As HTS technologies are heavily relying on bioinformatics analysis of the huge amount of generated sequences, it is of utmost importance that researchers can rely on efficient and reliable bioinformatic tools and can understand the principles, advantages, and disadvantages of the tools used. Here, we present a critical overview of the steps involved in HTS as employed for plant virus detection and virome characterization. We start from sample preparation and nucleic acid extraction as appropriate to the chosen HTS strategy, which is followed by basic data analysis requirements, an extensive overview of the in-depth data processing options, and taxonomic classification of viral sequences detected. By presenting the bioinformatic tools and a detailed overview of the consecutive steps that can be used to implement a well-structured HTS data analysis in an easy and accessible way, this paper is targeted at both beginners and expert scientists engaging in HTS plant virome projects.

## 1. Introduction

**Figure 1:**
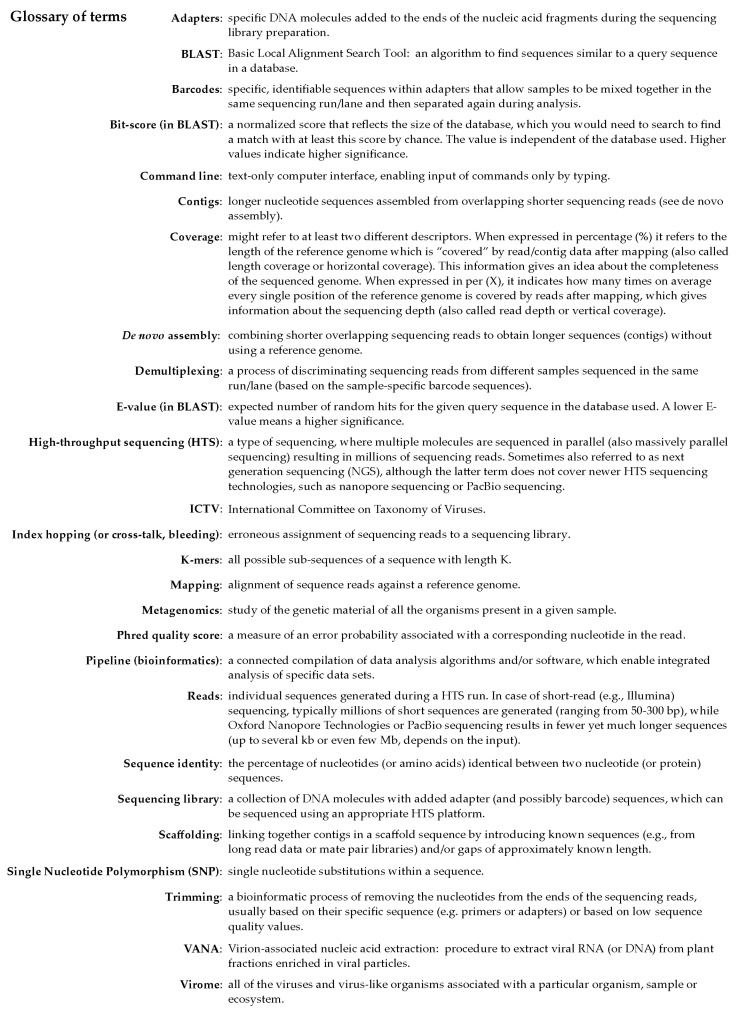
Glossary of terms commonly used in bioinformatics analysis of high-throughput sequencing (HTS) data for plant virus detection.

High-throughput sequencing (HTS) technologies have become an integral part of research and diagnostics toolbox in life sciences, including phytopathology and plant virology [1]. HTS enables the untargeted acquisition of extremely large amounts of sequence data from diverse sample types and thus represents an ideal and unique solution for the generic detection of highly diverse viruses. In the past decade, sequencing prices have significantly decreased, and the technology has become accessible to many more research and diagnostic labs. From the first uses of HTS for detection of plant viruses in 2009 [2,3,4,5], the use of this technology for detection of known and new plant viruses and the characterization of viromes in different plant species has intensified dramatically. Many different bioinformatics tools have been developed and different pipelines have been used to detect and identify plant viruses represented in HTS datasets. The variation in results associated with the use of different pipelines in different labs has highlighted the significance of understanding different approaches [6]. Arguably, one of the main challenges for less experienced users of HTS is to understand, select, and properly use tools for the analysis of HTS data intended for detection and identification of plant virus sequences. In this review, we aim to present the different and often complementary approaches used for analysis of HTS data for the detection of plant viruses. We provide a short introduction to the laboratory work required and then describe the possible steps in data processing for the detection of plant viruses, including quality control and trimming of the sequences, *de novo* assembly, sequence similarity searches, and taxonomic classification of the identified viral sequences. By including a short glossary (Figure 1), checklists, and comparison tables, we aim to present the topic to the widest possible audience and thus encourage the use of HTS technologies by researchers with limited experience in the field.

## 2. What Should I Anticipate and How Should I Prepare?

Modern sequencing platforms can generate massive amounts of data, and not all laboratories wishing to use HTS in their projects have the necessary infrastructure and bioinformatics expertise, which, for example, is one of the main challenges identified for the adoption of these technologies in diagnostic laboratories [7]. The cost of the bioinformatics analysis in a HTS project was estimated to be around 15% of the total cost of a program (an example for whole genome analysis in cancer research), and it includes the salary of the bioinformatician and cost of data storage [8].

Some commercial sequence analysis software is able to handle HTS data (see Section 4.3.8), with dedicated modules for common operations (e.g., mapping and assembly). These software solutions are usually easy to use, regardless of the user’s bioinformatics skill, but they are also quite expensive and might be limited for some analyses (specific applications). Furthermore, some “all in 1” viral-detection focused pipelines are available (see Section 4.3.8), which require only limited bioinformatics knowledge or only the help of a skilled computer scientist at the installation stage.

However, for in-depth analysis of plant virus sequence data that goes beyond detection and species classification, the use of dedicated bioinformatics software, without an easy-to-use graphical user interface, is often needed to optimize time and efforts. These programs have in a large part been developed and optimized for the Linux platform; they can be used in the command line only and so require specific computing skills. Considering the number of steps with the average HTS analysis pipeline and the number of samples studied, automation quickly becomes a priority. This can be achieved by writing scripts as well as grouping and ordering all the steps of the analysis, which also require expertise in programing languages (e.g., shell, Python, R). Finally, for the interpretation of the analysis results, skills beyond pure bioinformatics are needed. A close collaboration between a bioinformatician and a plant virologist (or a plant virologist trained in bioinformatics) is needed to achieve a meaningful interpretation of the results.

Beyond the skills of users, IT resources must also be addressed. The amount of data generated by each project must be anticipated in order to have raw data storage space available beforehand and to ensure that data is safely stored at least for several years after the end of projects. Depending on the sequencing platform, the total size of the raw data can become very large. For example, the Illumina NextSeq platform can generate from 120 to ≈300 Gbases (Gb) per run, leading to file sizes varying between 39 and 170 Gb depending on the read length. A stable and fast internet connection is often needed to facilitate the efficient transfer of large data files. The computing resources also need to be anticipated. For time-efficient analysis, it is often necessary to have a more powerful machine than an average workstation to run the various parts of pipelines, regardless of the software used. An alternative to the acquisition of a powerful computer is making use of online bioinformatics platforms and cloud computing solutions. These platforms generally have a structure adapted to the use of software making high demands on system resources (e.g., computing clusters). Many research centers or universities host a Galaxy instance, which represents a very good alternative to the Linux platforms, in a more “user friendly” interface.

## 3. Starting the Project: How Do I Prepare Samples and Sequence Nucleic Acids?

Sampling, nucleic acids extraction, viral enrichment, and sequencing library preparation are essential steps before HTS itself. Since these steps can influence the sequencing results, we briefly summarize here the most important considerations for some of these processes. An extensive description of how to control all of these steps is in preparation in forthcoming international guidelines for the use of HTS tests for the diagnostic of plant pests [9]. After obtaining the nucleic acids suitable for further analysis using HTS, the approximate amount of sequence data required per each sample should be estimated according to the goals of the study. If an external sequencing provider will perform HTS, this number, together with some general characteristics of the samples, should be communicated with the provider.

### 3.1. Input Material and Nucleic Acids Preparation

The extraction step separates the nucleic acids (including viral nucleic acids) from other cellular components. There are many methods that can be used to obtain high-quality nucleic acids intended for HTS [10,11,12,13]. The efficiency of an extraction method is evaluated by the quantity of nucleic acids obtained, their integrity, and the absence of contaminants that inhibit the enzymatic activities involved in the preparation of sequencing libraries. Irrespective of the chosen nucleic acid extraction procedure and library preparation methodology, it is recommended to collect several samples per plant or that tissue from distributed locations on a plant is combined into a single sample to overcome the uneven distribution of viruses, especially in the case of low titer viruses. Different types of nucleic acids can be used as inputs for HTS, which can be combined with different viral enrichment methods. No method is universal [11,14]; each favors certain viral families or certain experimental objectives [15]. For example, total RNA or small RNA sequencing might be most straightforward and universal to use for single samples. On the other hand, for sequencing of pools of many samples, or to optimize the detection of viruses with a low titer, methods that allow the enrichment of viral nucleic acids such as Virion-Associated Nucleic Acids extraction (VANA) or the purification of double-stranded RNA might be preferred. The choice for one of the approaches should be based on the research question and study design. The purpose of the following sections is to help make the most appropriate choices for sample preparation.

#### 3.1.1. Total RNA/DNA

Extraction of total RNA and/or, to a lesser extent, DNA is a widely used approach for HTS analysis of plant tissues infected with viruses. Simple and robust, the method can be carried out according to several standard extraction protocols in solid phase or in liquid phase or using commercial kits (mostly based on silica-membrane or magnetic bead purification). The extraction and sequencing of total DNA can be sometimes used specifically for the detection of DNA viruses, while sequencing of total RNA is a very generic approach and can be used for detection of all types of DNA and RNA viruses and viroids [15]. The high abundance of nucleic acids from the host plant co-extracted with viral nucleic acids can greatly limit the sequencing sensitivity. The relative proportion of viral sequences in the total extracted RNA can be increased by the depletion of the plant ribosomal RNA [16,17] and the proportion of sequences of circular DNA viruses in extracted DNA can be enriched by rolling circle amplification [18,19,20].

#### 3.1.2. Small RNA (sRNA)

The plant immune system responds to the presence of viruses by activating a defense response that leads to the cleavage of double-stranded forms of viral RNA into small RNAs (sRNA) of 21 and 22 nucleotides (nt) as well as, more marginally, of 24 nt [21]. The analysis of sRNAs facilitates the reconstruction of the complete genomes of infecting RNA and DNA viruses or viroids, as well as those of integrated endogenous viral elements (EVEs) if they are transcribed [2,15,22,23]. Since sRNAs are more stable than longer RNA molecules, the method is promising for use in old or even ancient plant samples [24], and since only very short reads are needed to sequence sRNAs, the method is relatively cost efficient. On the other hand, *de novo* assembly from short sequences might not work very well for targets present at a very low titer [15] and might lead to chimeric sequences in case of multiple infections with different virus strains [25]. For the same reason, pooled samples used in metagenomic studies including a large number of plants are not recommended to be analyzed with sRNA sequencing. Due to their short lengths, analyses of recombination events on a read level are also not feasible with sRNA [22].

#### 3.1.3. Virion-Associated Nucleic Acids (VANA)

The extraction of Virion-Associated Nucleic Acids (VANA) enriches the samples in nucleic acids of viral origin by semi-purifying the viral particles by ultracentrifugation. Viral particles are separated from most of the organelles and plant debris by one or two differential ultracentrifugation cycles depending on the viral family and the plant material. After purification of the particles and a nuclease treatment to degrade non-protected nucleic acids, the viral nucleic acids are extracted according to a standard extraction protocol also used for the extraction of total RNA/DNA. Initially developed for the biochemical characterization of viral particles in the 1970s, VANA was used in pioneering studies of prospecting for viral diversity in wild asymptomatic plants before the advent of HTS [26,27]. Then, the approach was extended to the preparation of nucleic acids intended for HTS [28,29]. It achieves balanced enrichment in high-quality viral RNA and DNA and allows the use of up to several hundred grams of starting material. However, it is based on the stability of the viral particles mainly determined by the pH and the concentration of salts in the extraction buffer. Unsuitable for high throughput, and relying on numerous laboratory operations, the approach only identifies the encapsidated viral nucleic acids as well as the viruses of the *Endornaviridae* family, which are devoid of capsids but encapsulated in membranous vesicles [28,30]. Moreover, certain viral families are difficult to purify, and VANA is also not the method of choice for the extraction of viruses from plants with high content of phenolic and polysaccharide compounds [31].

#### 3.1.4. Double-Stranded RNA

The majority of plant viruses have RNA genomes, accounting for 75% of the total number of viruses reported [32]. While plants do not produce large quantities of double-stranded (ds)RNAs, RNA viruses generate high molecular weight dsRNA structures during replication, so their enrichment is a popular strategy used for virus diagnostics [10,13,33,34]. The extraction of dsRNA purifies nucleic acids from double-stranded RNA viruses but also from most single-stranded RNA viruses, viroids as well as from some DNA viruses [35,36,37,38]. This approach allows the detection of a very wide range of RNA virus species [30,39]. Sequencing of dsRNA is likely not the most effective method for the detection of negative sense single-stranded RNA viruses [37]. It is also a laborious approach, even if a number of modified protocols have been developed to overcome this limitation [13,34,40,41,42].

### 3.2. Library Preparation and Sequencing

Following nucleic acid extraction, different methods have been developed for library preparation using commercially available kits and automated systems. As inputs, the extracted and possibly virus-enriched nucleic acids described in the previous sections can be used. The type of the library preparation and exact protocol is dependent on the input nucleic acids (e.g., total RNA or DNA, sRNA, dsRNA). Specific libraries are prepared for different HTS platforms. The library preparation step usually consists of fragmenting the nucleic acids and the ligation of short oligonucleotides (adaptors) at one or both extremities of the fragments in order to allow the sequencing. There are two main groups of HTS platforms: (i) short read HTS (also termed next-generation sequencing—NGS), producing reads up to several hundred nucleotides, and (ii) long read HTS (also termed single molecule sequencing—SMS), producing reads up to hundreds of kilobases (kb). Currently, the most commonly used sequencing platform is Illumina (short read HTS), and, for long read HTS, Pacific Biosciences (PacBio) and Oxford Nanopore Technologies. Nanopore sequencing is rapidly developing and is expected to be more widely used in the future [43]. Most of the available protocols recommend assessing the quality and quantity of the nucleic acids before library preparation. The integrity and purity of the nucleic acids can be assessed using spectrophotometric and fluorescence-based assays. For some enrichment approaches (e.g., VANA, dsRNA extraction), the concentrations of the obtained nucleic acids can be below the input required for library preparation so that a random amplification step may be required prior to library construction [13].

Several samples can be pooled and sequenced in the same sequencing run (multiplexing). In this case, the oligonucleotides ligated to the nucleic acids during library preparation also include unique barcode sequences that are specific for each sample. After sequencing, the reads are allocated to the appropriate sample according to the barcode used. Most commonly, the raw sequencing read data output is converted to a fastq file format. The fastq files represent an input for the bioinformatics analysis described in the following paragraphs.

Important consideration, when preparing samples for sequencing, is also, how many samples to pool in the same sequencing run/lane, i.e., how many reads (or nucleotides) are needed for the sensitive detection of different possible viruses in the plant sample. The answer is not straightforward, and it might depend on the sequencing approach, type of the matrix (host plant species, different parts of the plant), present virus(es), and other variables [15,17,38], such as, e.g., season, but also the sensitivity of the bioinformatics pipeline used (e.g., reads vs. contigs analysis) [6]. Some starting general recommendations regarding this problem are given in this primer; however, these need to be adjusted after performing a pilot study on a specific system, considering employed sample preparation, sequencing, and analysis approach.

### 3.3. Contamination

Contamination is common in all sensitive molecular diagnostic methods and has been reported in HTS diagnostics [44,45]. Contamination has been shown to enter sequencing systems in diverse ways, from sample cross-contamination [46] to external contamination of consumables [47]. Whilst some of the most commonly used HTS platforms from Illumina were subjected to significant hardware and procedural changes as a result of within-instrument DNA carry over, contamination can still be a significant issue in sensitive molecular diagnostics applications. The fundamentals of contamination control for diagnostics remain consistent. Key to achieving this is the separation of procedures into different locations, operating a one-way system (from clean reagents to DNA samples) within those locations and using negative controls at various stages to identify contamination. Sample-to-sample and reagent contamination are common in any molecular technique. Physically separating steps involving samples, purified DNA, and clean reagents is the best approach to preserve the integrity of future experiments. Known healthy control samples (not blanks), included from NA-extraction through to sequencing should be included in each run to identify incidences of contamination but are frequently excluded due to cost constraints.

## 4. How Do I Analyze the Data?

**Figure 2:**
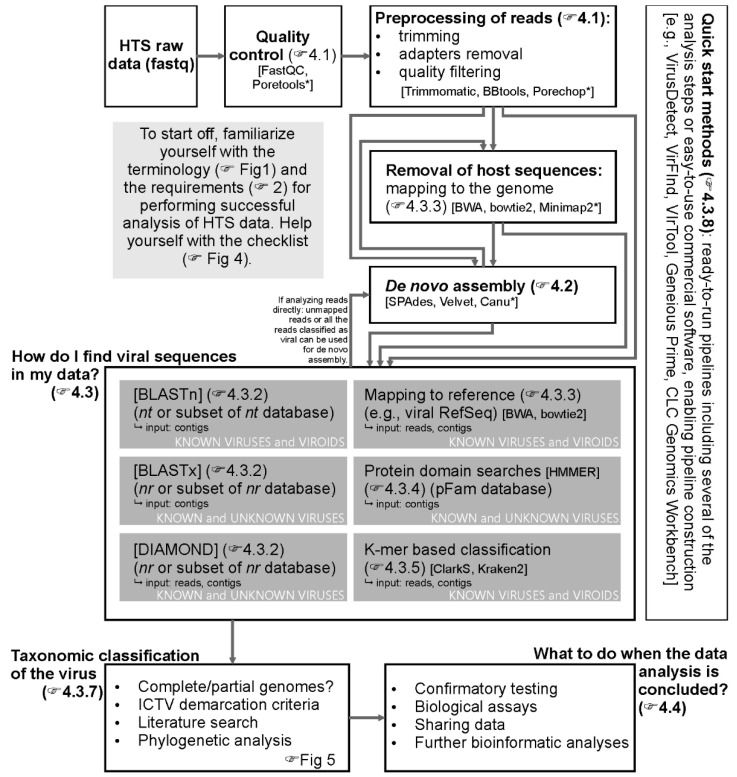
Flowchart representing different approaches for the analysis of HTS data for the detection of plant viruses. Boxes represent different steps in data analysis and interpretation. Arrows connect different possible sequences of the analysis steps. As an example, a non-exhaustive list of possible analysis tools is added in the square brackets at each of the analysis steps. Tools designated with * are intended for use with long-read or, specifically, nanopore sequencing data. Pointing hands lead to the text sections (or figures) with more detailed description of the corresponding steps.

Figure 2 outlines typical steps that can be followed once the fastq file has been obtained. The first is a quality control (QC) check. This is followed by pre-processing steps, including trimming low-quality bases, removing adapter sequences, and discarding very short and low-quality reads, followed by further QC filtering (Section 4.1). Then, reads passing QC are ready for analysis either directly or after assembly into contigs (Section 4.2). Reads or contigs can optionally be mapped to a host reference genome, and, in this way, host sequences can be removed (Section 4.3.3). Then, reads or contigs are used to query a database of known viral sequences or motifs (Section 4.3.2, Section 4.3.3, Section 4.3.4 and Section 4.3.5). Results need to be carefully inspected for correct taxonomic classification (Section 4.3.7). The described steps can be performed using the tools indicated in the flow chart (Figure 2) or other available tools. Finally, the same analyses can also be performed using user-friendly free software with graphical user interfaces (GUI) available online or using commercial software as described in Section 4.3.8.

### 4.1. Demultiplexing, Quality Control, and Trimming

Each sequencing platform produces a series of quality metrics associated with the data produced from each sequencing run. A discussion of the metrics with the sequencing data provider is important before accepting any sequencing data.

If the run was successful, the first step is the demultiplexing of barcoded samples, which is usually carried out using the sequencing platform software or performed by the sequencing data provider. In the event that data has not been demultiplexed, third-party tools such as Cutadapt [48] can be used to demultiplex the Illumina data by looking for specific barcode sequences present in the samples. Alternatively, demultiplexing tools developed by the sequencing platform provider are frequently accessible as stand-alone tools, such as Illumina’s bcl2fastq software [49], or Oxford Nanopore Technologies’ guppy scripts [50].

Barcode misassignments, also termed index hopping/cross-talk/bleeding, can occur due to the technical reasons during each sequencing run and result into erroneous assignment of a small fraction of reads from one sample to another one [51]. This represents a problem when using HTS for detection purposes, since it might often be difficult to distinguish index hopping from, e.g., very low titer virus infection in the sample. The amount of index hopping differs between different sequencing platforms, but it was, e.g., shown to be higher for newer Illumina sequencing devices using nonpatterned flow cells [52]. To mitigate this problem, it is advised to know the identity of all the samples sequenced in the same sequencing run or/and to use dedicated controls of the procedure. For example, including a control sample containing a known virus (which is not expected to be present in other samples in the run) could help estimate the amount of the crosstalk from the control sample to other samples, and vice versa. In addition, using unique double indexes in sequencing library preparation can largely reduce the amount of the index hopping [53].

Adapter sequences introduced during the library preparation process need to be removed. Tools such as Cutadapt [48], Trimmomatic [54], and Porechop [55] or NanoFilt [56] can be used to carry out this process, with the latter two working specifically for data generated using nanopore sequencers. At this step, contaminant filtering for synthetic molecules and/or spike-in is also recommended.

Sequencing data are usually provided in the fastq format, which consists of four lines per sequence [57], including a sequence identifier, raw nucleotide sequence, a separator line (containing + sign), and sequence quality values.

Nucleotides with a low-quality score should be removed to ensure that only high-accuracy bases remain. With Illumina data, values such as Q20 (1% error) and Q30 (0.1% error) are often used when trimming data, but this value depends on the application and the sequencing platform used. If accuracy is of the utmost importance (e.g., for detection of SNPs), selecting a higher quality score will be beneficial. If accuracy is less important (e.g., for detection of virus), then relaxing constraints on quality when trimming will allow more data to be available for downstream applications.

Quality control reports can be generated by tools such as FastQC [58], MultiQC [59], or, specifically for nanopore sequencing data, Poretools [60] or NanoStat [56]. This allows for the visual inspection of metrics such as per base sequence quality, sequence length distribution, and GC (guanine–cytosine) content. These reports can be generated both before and after trimming, to assess the impact of trimming on different quality parameters. A number of tools exist to trim sequencing reads based on quality scores, sequence length, or other metrics. These include but are not limited to Sickle [61], Trimmomatic [54], Cutadapt [48], BBDuk (https://sourceforge.net/projects/bbmap/, accessed on 13 April 2021) and NanoFilt for nanopore sequencing data [56]. Illumina data, particularly longer MiSeq reads, suffer from lower quality toward the 3′ end of the read. Many trimming strategies start at the 3′ end of such reads and determine the position at which the quality (or the average quality in a region) is high enough to keep.

The order in which these processes are carried out can vary, and some tools can be used to carry out multiple steps at the same time. The final output should be a series of demultiplexed samples with reads that have an acceptable sequence quality and no longer contain sequences added during the sequencing process (e.g., adapters, barcodes).

### 4.2. De Novo Assembly

HTS technologies provide us with shorter (e.g., Illumina) or longer (e.g., Oxford Nanopore Technologies, PacBio) sequence reads, which usually need to be assembled in silico to reconstruct complete or near-complete genomes. Compared to bacteria or eukaryotes, most viral genomes are very small. Nevertheless, high mutation rates and the great diversity of some viral populations [62] can represent a challenge for in silico genome reconstruction. Assembling a genome is similar to solving a “Jigsaw puzzle”. Similar to a puzzle, there could be pieces fitting together (overlapping reads), missing pieces (regions with low coverage, sequencing bias), and damaged parts (sequencing errors). The process for which individual reads are combined to form longer contiguous sequences is named *de novo* sequence assembly, and the nucleotide fragments obtained through this process are called contigs [63].

The intrinsic features of short vs. long read output, from the computational point of view, has led to the development of two major groups of assembly algorithms: (i) de Bruijn graph (DBG) and (ii) the overlap-layout-consensus (OLC) methods. In the first case, DBGs are constructed using k-mers, which are substring of the reads of length k; whereas for OLC, the overlap graphs are constructed directly from reads, eliminating the redundant ones. The use of k-mers is more widely applied for the assembly of short reads, whilst the OLC approach is most appropriate for long read data [63,64].

For short HTS reads, many de Bruijn graph assemblers are available, such as SOAPdenovo2 [65], ALLPATHS-LG [66], ABySS [67], Velvet [68], IDBA-UI [69], and (rna)SPAdes [70,71,72]. One of the first and most widely used and cited assemblers [73] in viral metagenomics [6] is the open-source software Velvet, which is followed by the more user-friendly and commercially-available CLC Genomics Workbench (https://digitalinsights.qiagen.com, accessed on 13 April 2021) and Geneious Prime (https://www.geneious.com, accessed on 13 April 2021). The latter has the advantage of providing a graphical interface for command-line assembly programs such as Velvet and Spades.

Different factors can positively influence the quality of the *de novo* assembly, e.g., a preliminary filtering step to eliminate the genomic host plant reads [23] or the selection of appropriate k-mer values based on the read length [6]. Moreover, approaches in which *de novo* assemblies using different k-mer values are generated and then reassembled can generally improve the completeness of *de novo* genome assemblies, but this can be a laborious and computationally lengthy process. Usually, higher sequencing depth and a higher fraction of viral reads in the dataset will positively affect the completeness of assembled viral genomes; however, extremely high coverage might have a negative effect on the completeness of the assembly when using some assemblers; thus, in such cases, the assembly of subsampled data might give better results [15]. Since reads of some viruses can be present in a very low number, it is important not to set too low cut-offs for contig length [6], e.g., a number around or slightly above the 2× length of an average read length is recommended. Finally, the use of an additional scaffolding step when using paired-end data can sometimes further increase the length of a contig. Nevertheless, despite improvements in *de novo* assembly algorithms, 3’ and 5´ ends of viral genomes usually cannot be obtained in full through *de novo* assembly.

Although long-read HTS platforms can produce reads close to full-length viral genomes, a major issue that could affect the *de novo* assembly step is the higher error rate (5–15%) of these technologies [74]. Long-read assemblers can algorithmically correct base errors before/when building contigs. PBcR [75], Canu [76], Falcon [77], and Pomoxis [78] are some of the OLC-based *de novo* assemblers available. Long read nanopore sequencing has recently been successfully applied to virus discovery, detection, and reconstruction of virus genomes; in these studies, Canu is the most cited assembler [79,80,81,82].

Contigs generated by *de novo* assembly can be used in subsequent similarity searches, and finally, viral contigs can be used for phylogenetic or recombination analysis. If this is so, it is important to check the quality of the contig by mapping the trimmed reads (explained in Section 4.3) to the viral contig followed by visual inspection of the mapping and to check the completeness of expected open reading frames contained in such contigs. For contigs generated by *de novo* assembly of nanopore sequencing reads, additional quality checking steps might be needed such as assembly polishing [81] or correction of the consensus sequences using quality data of mapping reads [82].

When the presence of specific viruses is already known, viral genomes can be reconstructed by mapping the reads (explained in Section 4.3) to the closest reference sequences obtained from sequence databases (after initial similarity searches, Section 4.3). Then, this is followed by the extraction of new consensus sequence from the mapping, which is an approach known as reference guided assembly. Sometimes, parts of the viral genomes are obtained by *de novo* assembly and other parts are obtained through reference guided assembly; such an approach is also known as combined assembly.

### 4.3. How Do I Find and Classify Viral Sequences in My Data?

Identification of viral reads/contigs in massive HTS datasets is most frequently performed by comparing sequences against known and annotated sequences in databases. This can be done on the level of reads or contigs *de novo* assembled from the reads. Since longer sequences in almost all cases improve the ability to identify similarities regardless of the method or databases used, an assembly of quality checked raw reads is generally recommended prior to similarity searches. At the same time, a prior assembly will also generally reduce the computing time needed for the similarity search steps, as up to millions of reads can be assembled in a single contig. The annotation of HTS reads, or contigs, on the basis of similarity with known viral sequences can be performed using three main strategies: homology searches with tools such as Basic Local Alignment Search Tool - BLAST [83], read/contig mapping against reference viral genomes using tools such as BWA [84], and the search for encoded, conserved protein motifs using tools based on Hidden Markov Models (HMMs) such as HMMER [85]. Each of these approaches and, in turn, each of the specific programs used to perform them, has advantages and drawbacks. In many cases, they should be seen as complementary rather than mutually exclusive possibilities. Several additional alternatives have also been proposed. For example, the use of e-probes (short unique pathogen-specific reference sequences) [86] or the analysis of the frequency of specific k-mer sequences (see Section 4.3.5). A summary of tools commonly used for similarity searches is presented in Table 1.

#### 4.3.1. Databases

The database(s) against which sequences are compared is/are of utmost importance for the efficiency and completeness of the annotation process. The more complete the collection of viral sequences, the greater the likelihood of detecting and identifying the presence of a virus. For BLAST and BLAST-like approaches, the most used databases are the non-redundant nucleotide database (nr/nt, named also just nt) hosted by the NCBI, the non-redundant GenBank protein database (nr) or the viral RefSeq database. The GenBank non-redundant nucleotide and protein databases are the most comprehensive and most frequently updated public databases, limiting the time from discovery of a novel virus to its availability for comparisons (provided the local version of these databases is also regularly updated). However, the size of these databases has the drawback of increasing the computing time/power needed to perform a comparison. The reduced viral RefSeq database has the benefit of a better annotation/curation at the expense of the number of included sequences and of less frequent updates. For read mapping approaches, smaller dedicated databases are generally used, such as a subset of all viral sequences from the NCBI nt database, viral RefSeq, or a smaller, locally developed and curated database (for example, one or several isolates of every virus known to infect the crop of interest). For conserved protein motifs searches, the most common databases are PFAM [87] and CDD [88]. The identification of viral sequences is critically dependent upon the quality of the database(s) used. For example, some plant-derived proteins might also be misidentified as viral if only a virus sequence database is used for similarity searches, because some viral proteins are related to plant encoded proteins. Typical examples are heat shock proteins (i.e., Hsp70h) found in closteroviruses [89] or reverse transcriptase proteins of *Caulimoviridae* that have homologs among retrotransposons. Wrongly annotated sequences in the public databases can also lead to erroneous annotations.

Although this is generally not implemented at the moment, comparing the identified viral sequences with databases of retrotransposons [90] or to databases created from the systematic screening of plant genomes for integrated viral sequences [91,92,93] may provide an efficient strategy to differentiate transcripts derived from integrated viral elements from autonomously replicating viruses.

#### 4.3.2. BLAST and BLAST-Like Approaches

BLAST programs are the most widely used and among the most accurate in detecting sequence similarity [94]. The BLAST suite [95] comprises different algorithms, each with its own use:

1. BLASTn can be used to compare a nucleotide sequence with a nucleotide database. It is less computationally intensive than BLASTx, but because of the higher divergence rate of nucleotide sequences, it is less efficient for the annotation of novel viruses not represented in the database used.
2. BLASTp can be used to compare a protein sequence with a database of protein sequences.
3. BLASTx can be used to compare a nucleotide sequence translated in all six reading frames with a database of protein sequences. While computationally intensive, it is the most efficient BLAST program for the annotation of novel viruses.
4. tBLASTn can be used to compare a protein sequence with all six possible reading frames of a nucleotide database and is often used to identify proteins in new, unannotated genomes.
5. tBLASTx can be used to compare all six reading frames of a nucleotide sequence with all six reading frames of a nucleotide database. It is the costliest in computation time.
6. MegaBLAST can be used to compare nucleotide sequences expected to be already present or closely related to those in a nucleotide database. It can be much faster than BLASTn and is able to handle much longer sequences but deals less efficiently with very divergent sequences.

Short sequences may lead to false positives in BLAST searches, and for this reason, other approaches should be preferred for very short reads or contigs. All BLAST programs return a table of results, which contain several parameters, among which some are particularly important to check: the identity threshold (threshold for the percentage of identical nucleotides between the query sequence and a hit in a database), e-value (expected number of random hits in the used database for a given query sequence), and query coverage (percentage of the query sequence covered by the database hit). It is very important to consider that some of these values depend on the size of the database used and that the use of too stringent parameters (e.g., identity threshold >85% and e-value smaller than 10^−10^) may lead to a failure to detect some divergent viruses [6]. BLAST is very widely used, but it remains, in the case of millions/billions of reads analyses, a time-consuming algorithm. Restricting the database used to specific taxa (e.g., viruses) can speed up BLAST searches, but care should be taken, as this frequently leads to the identification of viral reads that on closer examination, using complete databases, are in fact host sequences (e.g., plant sequences). An extremely fast but considerably less sensitive alternative to BLAST is BLAT (BLAST-Like Alignment Tool) [96]. Another faster alternative to BLASTx is DIAMOND [97], which runs at 500–20,000× the speed of BLAST while maintaining a high level of sensitivity, especially if using the sensitive mode. However, the DIAMOND annotations have been observed to be less optimal in virus species identification than BLAST ones (ML and TC personal observations).

#### 4.3.3. Mapping Reads (or Contigs) to Reference Database

Mapping tools are commonly used as a filtering step to remove host genome sequences or as a complement to similarity searches on short nucleotide sequences. Reads originating from the host genome can be partially removed by mapping the complete dataset to reference genomic sequences of corresponding host (if available) and then using only unmapped reads for further analyses. A reference genome sequence of the host must be chosen carefully, since it can affect the analysis. Choosing divergent variety/genotype of the host might reduce the efficiency of the host reads removal. Furthermore, reference host genomes might contain contaminating or genome-integrated viral sequences; thus, some viral reads can be lost in this step.

Mapping tools can be also used to perform the alignment of reads or contigs against a reference viral database (e.g., NCBI Viral RefSeq database or a custom developed database containing one or more complete or partial viral genomes). In comparison to BLAST programs, most of the mapping tools such as Bowtie2 [98] or BWA [84] build an index for the reference genome or the reads, increasing the speed of the analysis if used against a limited, virus-specific database. The mapping strategy is potentially more sensitive to detect viruses with low number of reads in analyzed datasets [6], in particular when using 21–24 nt sRNA sequences. Consequently, it is also sensitive to cross-sample contamination due to index-hopping, which may require the development of strategies to set a positivity threshold. On the other hand, mapping strategies are inefficient at detecting novel viruses or viroids that are absent from the database used. Mapping stringency parameters (see Table 1) critically affect the outcome of the analyses and should be optimized keeping in mind the objective of the experiment. Too stringent parameters may result in the failure to detect divergent viral isolates. Too relaxed parameters may also give rise to erroneous results through the mapping of related host genes on a viral genome or through cross-mapping the reads of a virus on the genome of a related virus. These problems can be minimized by first mapping all HTS reads against the reference viral database. Then, any reads that map to a virus are remapped against the host genome sequence. If the mapping score is higher for the host genome, the read is discarded. Tools such as Pathoscope [99] can help with cross-mapping between virus species by weighting reads that map to more than one viral sequence. An efficient strategy, besides counting the number of mapped reads on a particular reference genome, considers the portion of this genome covered by the mapped reads and depth of coverage, the percent similarity between mapped reads, and the reference or other similar indicators to eliminate potential false positive results. Including suitable reference samples as controls during sample preparation and sequencing can help to eliminate such errors [9]. Similar to reads, contigs generated by *de novo* assembly, can also be mapped to the reference databases. The longer the contig, the fewer erroneous mapping results are expected. However, the same recommendations for careful inspection of mapping results apply.

#### 4.3.4. Protein Domain Searches

Searching for known viral domains by matching translated amino acid sequences of reads/contigs with Hidden Markov Models (HMMs) of known protein domains using programs such as HMMER [100] or HMMScan is a popular alternative to BLASTx. With this method, sequences are first translated in all possible reading frames, and the translated protein sequences are compared to a database of conserved protein motifs such as Protein Families database—PFAM [87], viral profile HMMs—vFAM [101], and Conserved Domains Database—CDD [88]. These approaches are faster than BLAST-based homology searches and more effective than mapping or BLAST searches for the detection of very distant homologs [102] and therefore possibly for the detection of novel, very divergent viruses. Similar to BLAST, a significance e-value is calculated, allowing the evaluation of the significance of a match. This e-value can be used to filter results, striking a balance between low values and the reporting of false-positives, and high values and the failure to detect a divergent virus.

#### 4.3.5. K-mer Approaches and Machine Learning-Based Approaches

Nucleotide k-mer-based approaches can be used to annotate sequences based on the presence and frequency of specific k-mers. Comparing these frequencies is computationally less demanding and faster than sequence alignment but requires a lot of computer memory. Even if most of the k-mer-based classification tools, such as Kraken [103,104], Kaiju [105], or Taxonomer [106], are not dedicated toward the detection of plant viruses, they can be used for such purpose. Kodoja [107] uses a combination of such tools for the taxonomic classification of plant viruses in metagenomic data. Most of the tools are not very user friendly, and the use of k-mer tools for plant virus detection is fairly new; thus, some questions remain to be answered, e.g., the usability of k-mer tools on small RNA datasets [107].

Methods based on machine learning are being developed for the detection of viral sequences in metagenomics datasets. Several tools have already been published, e.g., ViraMiner [108], DeepVirFinder [109], or Virnet [110] for human virus detection purpose. Given a metagenome with known composition, machine learning approaches attempt to find some meaningful patterns that allow differentiating the host from the virus. When the unknown metagenome dataset is provided, the software should be able to discriminate virus sequences from host sequences using the learnt pattern. Machine learning tools are new in this field; thus, we still lack their in-depth comparison with the more known approaches discussed above.

#### 4.3.6. Which Analysis Approach Should I Choose?

**Figure 3:**
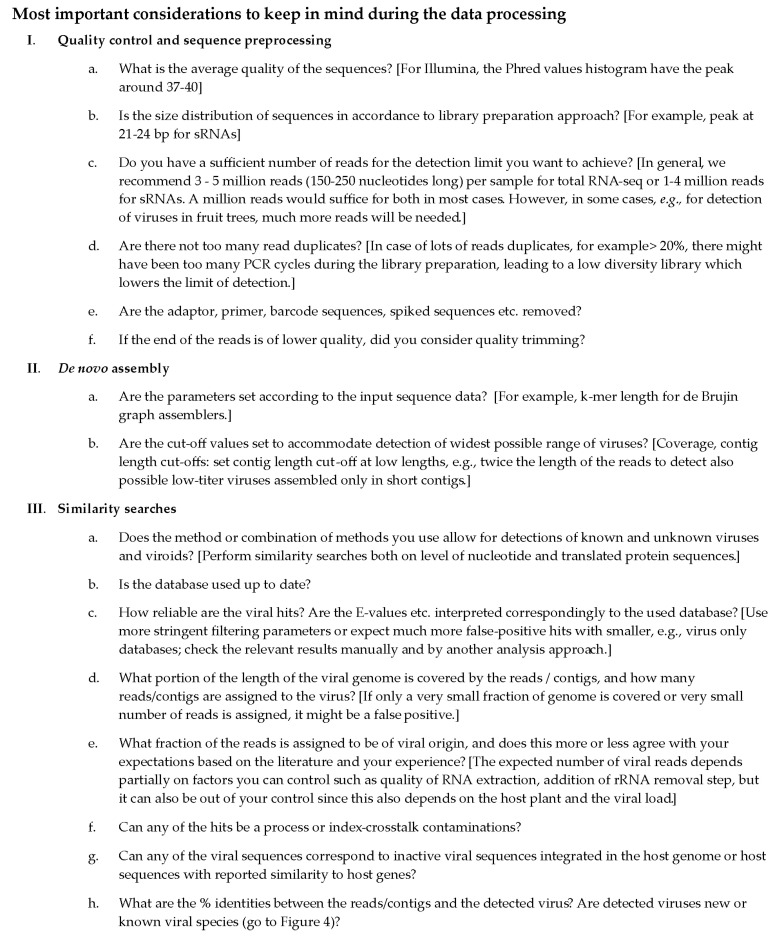
Checklist of the most important considerations to keep in mind during HTS data processing for detection of plant viruses.

The variety in similarity-based search approaches is striking. Choosing the most relevant one will depend on criteria such as the aims of the study (diagnostic, metagenomics) and the time/computational power available. Whichever program/approach is selected, it is important to consider its limitations and to properly set the key parameters to avoid false-positive or false-negative results. Fast programs can be used as a filtering step and then validated by slower approaches, or alternatively, two approaches can be used to validate each other, or multiple approaches can be used in parallel, for example an optimized approach for the detection of known viruses and a separate approach for novel virus discovery. If computational time or power is not a serious limitation, combining several approaches may enhance the ability to obtain an accurate annotation [111]. Here, we provide a checklist, identifying the most important considerations, which should be taken into account when analyzing HTS data (Figure 3).

Moreover, when analyzing the data obtained from long-read technologies, one should pay special attention to using approaches that enable the efficient processing of such data. Mapping algorithms have been developed for the processing of long read data with higher error rates, such as Minimap2 [112]. For BLASTx-like similarity searches, algorithms that can handle frame-shift mutations (caused by the relatively higher error rates), such as DIAMOND [97], are preferred. Assembly and polishing of long read data can improve further processing [113] and improve the chances for the correct identification of viral sequences in the data.

#### 4.3.7. Taxonomic Classification

To assign viruses to taxonomic ranks, demarcation criteria specifically set for different viral genera need to be followed. Often, identities <75% at the nucleotide or protein level are indicative of a new viral species; however, the threshold might be also lower or higher, such as at <91% for begomoviruses. Identities <60% might be indicative of a new viral genus; however, the threshold might be also lower or higher, such as <45% within *Betaflexiviridae* family. As noted, these criteria differ substantially between virus families and genera, but up-to-date information is published by the International Committee on Taxonomy of Viruses (ICTV) in the latest taxonomy reports [114,115] that can be found online (https://talk.ictvonline.org/taxonomy/, accessed on 13 April 2021). Once a sequence is identified to a family or genus level, a pairwise sequence comparison (PASC) webtool [116] to support virus classification, hosted by NCBI (https://www.ncbi.nlm.nih.gov/sutils/pasc/, accessed on 13 April 2021), can quickly provide an indication on how a new sequence fits in that genus or family. In cases where virus sequence identity is near the limit of the identity cut-off values for different species, additional information and/or justification may be required for their definite classification. These could include biological information such as host species, vector species, or symptom types, and if enough isolates have been sequenced, population genomics approaches can also be employed [117].

Strains of viruses do not fall under official taxonomy. Rather, they are definitions utilized by communities of practice around virus species and would thus require a review of the literature concerning the specific virus species to be able to classify the sequence to a particular strain or phylotype. This is a process that generally includes phylogenetic analysis of the identified sequence with published virus (reference) sequences.

**Figure 4:**
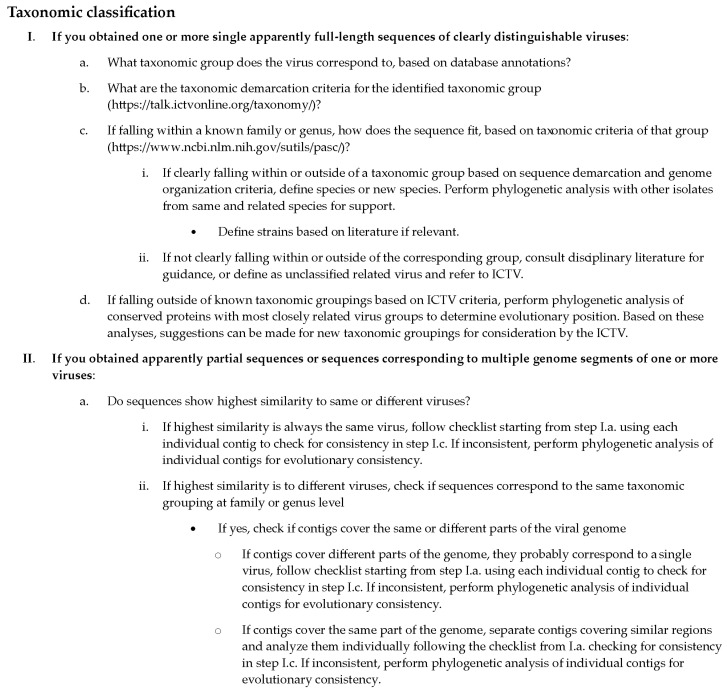
Checklist of the most important considerations during taxonomic classification of plant viruses detected by HTS.

The approach described above can be rather straightforward if complete genomes of viruses with a single genome segment have been assembled. However, things can become more ambiguous in situations where a new virus has multiple genome segments or have been incompletely assembled, resulting in several contigs corresponding to different parts of a viral genome. The individual contigs for a novel virus may be equally distantly related to several known viruses and can then show the highest level of similarity with different viruses, which could lead to the erroneous interpretation that several new viruses are found in the same sample. This issue will often manifest itself in the previous step of similarity searches, and, to resolve this, the first recommended step is to identify the taxonomic position of all the best hits identified for the different viral contigs. If several best hits fall within the same genus or family, one could suspect they may correspond to the same virus. The next step would be to investigate the general viral genome structures in the identified genus or family from the ICTV reports and ascertain if the different best hits correspond to the same or different genomic regions for that type of virus. If they are all different, it is likely that a single new species is present; if the same region is covered by multiple contigs that differ significantly from each other, then the scenario of multiple new viruses belonging to a similar taxonomic group is more probable. A checklist in Figure 4 contains the most important points to keep in mind for the taxonomic classification of viral sequences obtained by HTS.

Sequences of new viruses belonging to previously undescribed families and/or genera can often only be reliably aligned by using the translated amino acid sequences of conserved genes such as polymerases and coat proteins. In these cases, phylogenies generated with viruses from related genera or families are needed to determine the exact taxonomic position. Additional criteria, such as number of open reading frames and overall genomic organization, need to be considered when classifying a virus as a member of a new genus or family. When there is uncertainty, viruses can be categorized as unclassified new species until new evidence arises that can support a definite classification.

Irrespective of the situation encountered, to become an officially recognized new species, generally, a near complete genome sequence, including the complete coding sequence information, is required by the ICTV to assign a “sequence only” virus to a species level. If relevant supportive biological data are available, that rule is more relaxed and will be determined by the relevant virus family study groups.

#### 4.3.8. “Quick Start” Methods

**Figure 5:**
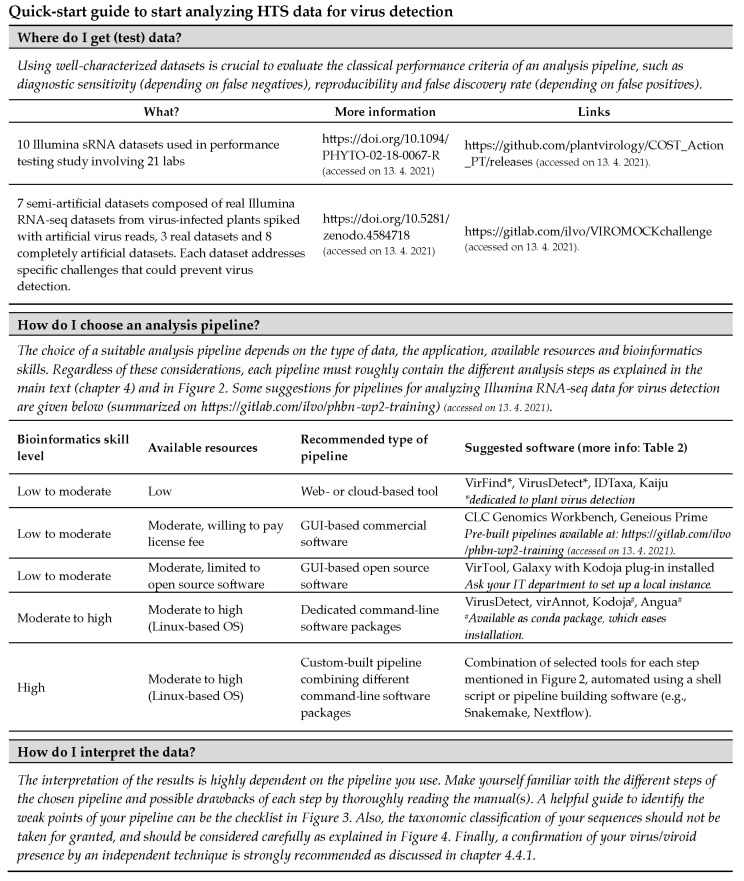
Quick-start guide assisting selection of analysis approaches for plant virus detection from HTS data.

Depending on the computational background of the user, there are different ways to approach the analysis. Many software solutions are available for detecting the presence of (plant) viruses in HTS datasets, which have been summarized recently by several reviews [118,119]. For beginners or newcomers in the field, all these tools can be overwhelming. The quick-start guide (Figure 5) might be handy to select an appropriate tool or pipeline.

Among these options, easy-to-use pipelines that do not require extensive computational expertise might be a good start. These pipelines present a user-friendly interface on-line or directly on the computer. A first group of pipelines can be considered as “all in one”: they automatically start on the raw data to deliver the final results as a list of viruses detected. They may or may not allow the adaptation of parameters. A second group corresponds to pipelines for which the different steps of the process have to be done separately and independently. This is the case when using commercial software such as CLC Genomics Workbench or Geneious Prime, which both also enable the building of customized “all-in-one” workflows. Table A1 summarizes the pros and cons of the most common “easy-to-use” analysis solutions. Ease of use may generate a false sense of confidence in the results and, as with all pipelines, understanding of the steps and the parameters of the pipelines, as well as critical interpretation of the results is always required.

### 4.4. What to Do When The Data Analysis Is Concluded?

#### 4.4.1. Identity Confirmation by an Independent Technique

As for many other test methods, HTS may sometimes provide false-positive results. Therefore, if consequential, it is important that HTS results are confirmed.

The need to confirm the identity of a pest depends on the context of the analysis and on the type of organism identified (e.g., identification of a quarantine compared to an endemic pest). The results must be confirmed in cases considered critical to national or international plant protection programs. These are the detection of a pest in an area where it is not known to occur or in a consignment originating from a country where it is declared to be absent; and also, when a pest is identified by a laboratory for the first time (EPPO PM 7/76, 2019). The identity of any uncharacterized pest with potential risks to plant health should also be confirmed by another test. Whilst a virus in its common host is unlikely to require confirmation (if not regulated), it may be useful if associated with different symptoms (e.g., an emerging strain).

When confirmation is needed, it is recommended to use a test or a combination of tests based on different biological principles (e.g., ELISA or targeted PCR instead of resequencing the sample using the same protocol). If available, validated tests should be used and a new sample extract obtained for analysis. The selection of confirmatory tests depends on the performance characteristics required; the general characteristics of methods for plant virology have been reviewed [120]. If no other tests are available to confirm the identity of the pest (i.e., poorly characterized and uncharacterized organisms), primers should be designed and tested, based on the HTS sequence data and available sequence information in the sequence databases. Alternatively, generic primers that enable the amplification of viruses within a genus or family, including the targeted one(s), followed by Sanger sequencing of the amplicons could be used to confirm the identity.

#### 4.4.2. Biological Characterization Post HTS Detection

Based on HTS, the list of thus far unknown or poorly characterized viruses for which only genome data are available is rapidly increasing [121]. This presents a challenge for the further steps necessary to determine the causative relationship to a disease and guide phytosanitary diagnostic laboratories on data interpretation and recommendations. Viruses for which only genome data are available can indeed be taxonomically assigned, but the real challenge is to attribute biological meaning to their detection. The interpretation of the biological relevance applies mainly to poorly characterized and uncharacterized or newly discovered viruses. For example, the viral sequences detected may correspond to a *bona fide* virus infecting other organisms associated with the sample, including bacteria, fungi, or arthropods [122,123] or to viral sequences integrated into the plant genome [124,125]. As stated previously [125], relevant scientific expertise is essential for sound biological interpretation of HTS results, in particular when identifying a target with a low titer, a poorly characterized species, an uncharacterized organism, or sequences integrated in the host genome [6,126]. In this latter case, careful phylogenetic analysis, including retrotransposons and viruses reported only from integration events in plant genomes [91,92,93] may provide critical information on whether the sequences identified correspond to an autonomously replicating (episomal) virus or to cellular transcripts from integrated viral elements. This may need to be validated by specific experiments to confirm or disprove an episomal replication scenario.

The extent to which additional biological characterization is performed depends largely on the potential risk the organism(s) would pose to plant health, although the acquisition of such data may take time or may not be possible (e.g., lack of human and/or financial resources). The scaled and progressive scientific framework proposed by Massart et al. [125] is a useful tool for guiding the biological characterization and the risk assessment of an uncharacterized or poorly characterized plant virus detected by HTS.

#### 4.4.3. Sharing Data to Leverage Knowledge

After the detection of the virus in the laboratory, the researcher or diagnostician faces an important dilemma: when and how to share data publicly. As shown by recent examples [127,128,129], pre-publication data sharing between laboratories brings valuable information to address the risks raised by a virus. Sharing data will give a more global picture of its geographical repartition, its genetic diversity, its host range and symptomatology, allowing a contextualized risk analysis and avoiding unnecessary regulatory action. When shared, the genome information usefulness is leveraged. Data sharing must also include metadata from the sample (e.g., origin, species, cultivar, time point, organ of sampling). Nevertheless, data sharing is not always easy due to regulatory implications, and for commercial work, laboratories may be bound by confidentiality agreements [7]. In addition to sharing sequence data itself, sharing of analysis pipelines, protocols, and experiences between labs can greatly contribute to the harmonization of the field and provide useful resources for newcomers to the field. The recently established Plant Health Bioinformatics Network (PHBN) aims to foster this approach and provide protocols, pipelines (https://gitlab.com/ilvo/phbn-wp2-training, accessed on 13 April 2021), and reference datasets (https://gitlab.com/ilvo/VIROMOCKchallenge, accessed on 13 April 2021) [130] that can be widely employed. It also aims to organize community efforts to advance certain aspects of plant health bioinformatics (https://gitlab.com/ilvo/PHBN-WP4-RNAseq_Community_Screening, accessed on 13 April 2021).

#### 4.4.4. Recombination Analysis

Recombination is common in some genera of plant viruses, and the presence of recombination events can have impacts on downstream analysis such as phylogenetics. Thus, identification of recombination is a useful first step, prior to further genome analysis. The most popular software solutions, which detect recombination patterns comparing full or partial viral genomes and run on Windows, are RDP4 [131], SimPlot [132], and TreeOrder Scan [133]. ViReMa (Viral Recombination Mapper) can be used for the detection of recombination junctions, as well as insertion/substitution events and multiple recombinations within single reads [134], and it has been successfully applied for the analysis of recombination events in plant virus genomes [22,135,136].

#### 4.4.5. Additional Bioinformatics Analyses

Further analyses, beyond viral detection and taxonomic classification, can be performed on HTS data, depending on the goal of the study. For instance, the large amount of sequence data generated by HTS allows a good resolution of the within-host genetic diversity of the viral populations [22]. Assessing the genetic diversity within and among viral populations can provide a better understanding of virus evolution and help to determine population genetic parameters or epidemiological patterns [137,138]. This can be done using single nucleotide polymorphism (SNP) calling algorithms, which need to allow the detection of low-frequency variants expected in virus populations. Phylogenetic relationships among the detected and previously known viruses can also be investigated using fast neighbor-joining algorithms [139], more precise maximum likelihood approaches [140,141], or Bayesian analysis approaches [142]. Freeware phylogenetic analysis suites, such as MEGAN [143], or phylogenetic analysis algorithms integrated within commercial software, such as CLC Genomics Workbench and Geneious Prime, can be used. Studying the time of emergence of viral species and strains including the distribution of the genetic diversity across geographical sites can be done using software such as BEAST [144], TempEst [145] and SPAGeDI [146].

## 5. Conclusions and Outlook

In this review, we aimed to provide an informative primer on the generation and analysis of HTS data for the detection of plant viruses. Even though the field of HTS is transforming rapidly and new platforms and analysis tools are being developed constantly, the basic concepts of data analysis reviewed here will remain relevant in the future. In the next few years, we expect a great increase in the use of the long-read HTS platforms. New algorithms and pipelines for analysis of data will continually be developed, building on some of the concepts described above. These developments are likely to focus on two main areas. Firstly, the adoption of deep learning approaches will likely be more and more integrated into the field of virus detection, on different levels, from similarity searches to the estimation of detection confidence levels, to enable the more robust detection of virus sequences that are more distantly related to those we currently recognize. Secondly, with the further development of nanopore sequencing-based platforms, potentially facilitating on-site HTS analysis of samples, we will need faster and more memory-efficient analysis approaches to enable rapid data analysis, potentially away from centralized facilities. Moreover, guidelines are being developed to enable the validation and verification of HTS-based detection of plant pathogens in research and diagnostic settings, which also include bioinformatics steps of the analysis [9]. These guidelines will provide detailed information on how to use appropriate controls and which specific results parameters to use to ensure the validity of the results, which is briefly covered in Figure 3 and Figure 4 in this text. Finally, we encourage the readers to use this guide as a starting point for the selection of appropriate analysis approaches and to get further informed about the specifics of the algorithms (Figure 5). By combining knowledge on the analysis approaches with a sound plant virology background, we can maximize the potential of these technologies and provide sound interpretation of the results.

## Figures and Tables

**Table 1:** Summary of the most commonly used similarity search strategies with advantages and limitations for each of the strategies.

Tool Name	Advantages	Limits and Considerations	Important Thresholds
BLASTx or BLASTn	High sensitivity	Slow, intensive use of computing power if a large database is used, BLASTx needed for the detection of divergent novel viruses, BLASTn needed for the detection of viroids and noncoding regions of viral genomes or satellites; performance improved by prior assembly of contigs.	Minimum percentage of identity; length of identified region of similarity; minimal e-value, bit-score.
MegaBLAST	Faster than BLASTn, handles longer sequences	Less sensitive than BLASTn, only useful for detection of nucleotide sequences very similar to the ones in the used database; performance improved by prior assembly of contigs.	Minimum percentage of identity; length of identified region of similarity; minimal e-value, bit-score.
BLASTp	High sensitivity	Slow, need to translate nucleotide sequences to proteins first; performance improved by prior assembly of contigs; not applicable for viroids or noncoding regions of viral genomes or satellites.	Minimum percentage of identity; length of identified region of similarity; minimal e-value, bit-score.
DIAMOND	Faster than BLASTx	Less sensitive, annotation less accurate than BLAST; performance improved by prior assembly of contigs; only available for searches against protein databases; not applicable for viroids or noncoding regions of viral genomes or satellites.	Minimum percentage of identity; length of identified region of similarity; minimal e-value, bit-score; use sensitive mode.
Burrows-Wheeler transform-based mapping algorithms (e.g., BWA or Bowtie2)	Does not require prior assembly of contigs, high sensitivity for short sequences	Only allows detection of known agents. Difficult to adjust mapping stringency to (1) allow detection of divergent isolates while (2) avoiding cross-mapping between related agents; prior assembly of contigs reduces cross-mapping between related agents.	Mapping stringency (e.g., mismatch penalties, gap open/extension penalties, percent of read length matching reference, minimum percentage of identity)
HMMER or HMMScan	High efficiency for detection of distant homologs	Annotation more complex for protein families shared between cellular organisms and viruses; not applicable for viroids or noncoding regions of viral genomes or satellites.	Minimal e-value.
K-mer based classification algorithms (Kraken or Taxonomer)	Fast	Requires large computer memory; accuracy may be limited for the shorter genomes of plant viruses; the confidence scoring of the results is not straight forward.	C/Q ratio for Kraken (advise the manual).

## Appendix A

**Table A1:** List of selected easy-to-use analysis solutions for detection of plant viruses with their pros and cons.

Pipeline	Brief Description	Web Link/Publication	Pros	Cons
**VirusDetect**	Virus discovery using sRNA and RNAseq sequences	http://virusdetect.feilab.net, accessed on 13 April 2021 [147]	• Easy to use: single command to run one or multiple datasets simultaneously. • Performs *de novo* assembly and reference mapping in parallel, including optional host genome subtraction and identified contigs through BLASTn and BLASTx. • Automatic results organization and presentation in html table providing key metrics on coverage, sequence depth, virus and genus name, and link to visual map and NCBI GenBank reference sequence. • Options to modify key assembly, mapping, and reporting parameters. • Windows version with visual interface and automatic quality control and trimming to be released in 2021. • Available via user account online.	• Uses complete NCBI GenBank database for viruses (divided along host type) for reference mapping and identity searches. NCBI GenBank sequences are poorly curated and may lead to reports of wrong results. • Creating and formatting new custom or up-to-date NCBI GenBank reference library is not very straightforward and ready formatted updates are not uploaded very regularly to the VirusDetect webpage. • Currently requires Linux environment, which is an impediment for many diagnosticians. • Default reporting cutoff settings are optimized for siRNA to minimize false positives due to index-hopping; however, they may lead to the non-reporting of low concentration viruses.
**Virtool**	HTS sample manager with virus detection, discovery and analysis workflows	www.virtool.ca, accessed on 13 April 2021 https://github.com/virtool/virtool, accessed on 13 April 2021 [36]	• Open source modern graphical optimized for cloud computing. • User and group control with password protection, sample data management, security, and QA features. • Support for multiple workflows and versioned databases for viral and non-viral pathogens. • Can process short and long reads (Illumina). • Result visualization, filtering, and sorting. • HTTP API for automation or integration with other services such as LIMS. • Can also be controlled via the command line for more complex tasks.	• Requires some computational skills for user (or help of informatician) to install as a local server on Linux operating system. • Limited ability to change parameters within a workflow.
**virAnnot**	Command-line tool for virus detection and viral diversity estimation	[148]	• Wide options to modify assembly, mapping, annotation, and clustering parameters. • Performs parallel analysis of samples from the same dataset. • Estimation of viral diversity through Operational Taxonomic Units (OTUs). • Easy results visualization with Krona and phylogenic trees.	• Requires a Linux environment, which is an impediment for many diagnosticians. • Need a cluster access for the annotation step. • Requires a good knowledge of command-line and Unix packages installation.
**VirFind**	Online virus discovery tool	http://virfind.org, accessed on 13 April 2021 [149]	• Available via user account online. • Performs reference mapping, *de novo* assembly, and conserved domain searches in parallel or subsequently.	• Analysis by online version can take several days. • Output only in text files: experience needed for further interpretation.
**Angua**	Command-line tool for virus detection	https://fred.fera.co.uk/smcgreig/angua3, accessed on 13 April 2021	• Simple: can be executed with one command but has a number of parameters/tools that can be tweaked • Uses full nt and nr GenBank databases so is sensitive • Manual inspection of results with a local MEGAN installation improves accuracy • Supports single and paired-end analysis • Supports BLASTn/MEGAN parallelization	• Requires a Linux environment, which is an impediment for many diagnosticians. • Dependent on locally stored nt and nr GenBank databases. • BLASTx stage can take a long time. • Manual inspection of results with a local MEGAN installation is required.
**Kodoja**	k-mer based command-line tool for virus detection	https://github.com/abaizan/kodoja, accessed on 13 April 2021 [107]	• Available as Galaxy plug-in or as command-line tool that can be installed using conda. • k-mer based rather than assembly and mapping, which makes it more sensitive and computationally less intensive.	• Requires a Linux environment for the command-line tool, which is an impediment for many diagnosticians.
**Truffle**	Targeted virus detection using e-probes based approach	[150]	• Results easy to interpret, good sensitivity. • Requires relatively low computational resources.	• Undescribed virus or viral strain will not be detectable using this pipeline. • Only grapevine and citrus viruses are available; however, e-probes for other viruses can be designed. • Requires a Linux environment, which is an impediment for many diagnosticians.
**Kaiju**	Online metagenomic analysis tool	http://kaiju.binf.ku.dk/, accessed on 13 April 2021 [105]	• Both standalone and web server available. • Quick analysis not requiring any knowledge in bioinformatics and data analysis. • Prepared downloadable databases available.	• Not specifically made for virus detection. • Protein based, hence blind for non-coding sequences (viroids, satellites).
**Galaxy**	Workflow system for computational analyses	https://usegalaxy.org, accessed on 13 April 2021 [151]	• Web-based platform. • Open source. • Vast choice of computational biology tools.	• Limit in data upload, unless if you establish own local galaxy server. • Not specifically made for virus detection.
**ID-Seq**	Online metagenomic analysis tool	https://idseq.net/, accessed on 13 April 2021 [152]	• Easy-to-use visual interface of results. • Quick analysis not requiring any knowledge in bioinformatics and data analysis.	• Not possible to change parameters of the workflow. • Complementary software needed for reads alignment. • Not specifically made for virus detection.
**Geneious Prime**	Software for molecular biology and sequence analysis	https://www.geneious.com, accessed on 13 April 2021	• Graphical interface. • Multiple plugins available, including some frequently used freeware assembly algorithms. • Automated, customizable workflows. • Constant release of updated versions and customer support. • Nice and efficient visualization tools. • Free trial version available.	• Licensed, including license fee; • HTS data analysis requires computational resources.
**CLC Genomics Workbench**	Comprehensive software solution of molecular biology analysis tools	https://digitalinsights.qiagen.com/products-overview/discovery-insights-portfolio/analysis-and-visualization/qiagen-clc-genomics-workbench/, accessed on 13 April 2021	• Graphical interface. • Automated, customizable workflows. • Constant release of updated versions and customer support. • Nice and efficient visualization tools. • Free trial version available.	• Expensive ongoing licensing fee. • HTS data analysis requires computational resources.
